# Coexistent Hashimoto’s thyroiditis modulates lymph node metastasis burden in papillary thyroid carcinoma: a single-center study in South China

**DOI:** 10.3389/fendo.2026.1929155

**Published:** 2026-08-20

**Authors:** Tianyu Gan, Dongyuan Gan, Guansheng Liao

**Affiliations:** 1 Department of Thyroid Surgery, Huizhou Central People’s Hospital, Huizhou, China; 2 Department of Obstetrics, Dongguan Songshan Lake Central Hospital, Dongguan, China

**Keywords:** Hashimoto’s thyroiditis, lymphatic metastasis, neoplasm staging, papillary thyroid carcinoma, risk factors

## Abstract

**Objective:**

To investigate the impact of Hashimoto’s thyroiditis (HT) on central lymph node metastasis (CLNM) patterns and identify independent risk factors for CLNM in patients with papillary thyroid carcinoma (PTC).

**Methods:**

This retrospective study enrolled 668 cN0 PTC patients who underwent initial surgery at Huizhou Central People’s Hospital between August 2024 and July 2025. Patients were stratified into an HT-PTC group (n = 117) and a PTC-only group (n = 551) based on postoperative histopathological findings. Clinicopathological features and CLNM characteristics were systematically compared. Multivariable logistic regression was utilized to identify independent risk factors for CLNM in patients with HT-PTC, and the predictive efficacy of the resulting model was validated using ROC curves.

**Results:**

The HT-PTC group exhibited significantly higher rates of multifocality and central lymph node dissection, alongside lower BMI and serum thyroglobulin (Tg) levels, compared with the PTC-only group (all *P* < 0.05). While the incidence of CLNM did not differ significantly between groups (35.0% vs. 33.9%, *P* = 0.819), the metastatic lymph node ratio (LNR) was significantly lower in the HT-PTC group (*P* < 0.001). Multivariate analysis identified tumor diameter > 1 cm as an independent risk factor for CLNM among HT-PTC patients (OR = 3.678; 95% CI: 1.587–8.520, *P* = 0.002). The predictive model demonstrated moderate efficacy (AUC = 0.649). Notably, the incidence of CLNM in HT-PTC patients with microcarcinoma (≤ 1 cm) was only 25.9%.

**Conclusion:**

Concomitant HT is associated with a reduced metastatic burden rather than an increased prevalence of CLNM in patients with PTC. Tumor diameter > 1 cm serves as an independent predictor of CLNM. For HT-PTC patients with tumors ≤ 1 cm and no other high-risk factors, the necessity of prophylactic central lymph node dissection warrants individualized assessment combining clinical data and intraoperative observations to avoid overtreatment.

## 1 Introduction

The global incidence of thyroid malignancy has increased steadily over the past three decades, currently ranking as the most prevalent malignant neoplasm of the endocrine system (1). In China, thyroid cancer is the most common endocrine malignancy in females aged 15 to 39 years (2), with higher age-standardized incidence rates in South China relative to the national average (3). Papillary thyroid carcinoma (PTC) accounts for 85%–90% of all confirmed thyroid cancer cases (4). Despite the generally favorable overall survival (OS) of patients with PTC, cervical lymph node metastasis remains a critical determinant of disease recurrence and clinical prognosis. Specifically, the incidence of central lymph node metastasis (CLNM) in PTC patients ranges from 30% to 80% (5, 6). CLNM has been established as an independent predictor of postoperative local recurrence, reoperation, and disease-specific survival (DSS) in PTC populations (7).

Hashimoto’s thyroiditis (HT) is the most prevalent autoimmune thyroid disorder, with an overall adult prevalence of 5%–10% (8). Females are 7 to 10 times more susceptible to this condition than males (9). Histopathologically, it is characterized by massive lymphocytic infiltration and follicular cell damage within the thyroid gland (10). Epidemiological data demonstrate that HT is more frequently identified among PTC patients than in the general population, which has spurred extensive research into their intrinsic correlation (11). However, substantial debate remains regarding how HT affects the oncogenesis, progression, invasiveness, and clinical outcomes of PTC.

Available studies report contradictory effects of HT on PTC biological behaviors. Patients with concomitant HT and PTC often exhibit favorable clinical outcomes, including lower lymph node metastasis rates, decreased extrathyroidal extension, and improved disease-free survival (DFS) (12–14). Conversely, chronic inflammation associated with HT may accelerate tumor development and increase the risks of multifocality and bilateral disease, positioning HT as a potential predisposing factor for PTC (15). Alternatively, HT-related immune infiltration and lower rates of BRAF V600E mutations may suppress tumor progression and produce an indolent tumor phenotype (16, 17). Discrepancies among studies may stem from differences in geographic regions, races, iodine nutrition, and HT diagnostic criteria. Few studies have explored CLNM characteristics and risk factors in HT-complicated PTC patients in South China, a region characterized by varying population iodine levels despite being iodine-sufficient.

Prior investigations generally utilized CLNM status as the primary study endpoint and rarely addressed the metastatic lymph node ratio, a more robust marker for reflecting tumor burden. Using a large single-center surgical cohort of PTC patients in South China, we compared clinicopathological characteristics between patients with and without concomitant HT. We further analyzed HT-related alterations in CLNM patterns and determined independent risk factors for CLNM in patients with concurrent HT and PTC. These findings may facilitate the development of individualized treatment strategies for PTC in this region.

## 2 Materials and methods

### 2.1 Study population

This retrospective analysis enrolled 668 patients diagnosed with PTC who underwent surgical intervention at the Department of Thyroid Surgery, Huizhou Central People’s Hospital (a coastal iodine-sufficient tertiary center in South China), between August 2024 and July 2025. The study protocol was approved by the institutional Medical Ethics Committee and conducted in accordance with the ethical guidelines of the Declaration of Helsinki. Informed consent was waived due to the retrospective nature of the study.

Inclusion Criteria: (1) Patients undergoing primary thyroidectomy accompanied by systematic central lymph node dissection; (2) Postoperative pathological diagnosis of papillary thyroid carcinoma; (3) Negative findings of central lymph nodes on preoperative ultrasonography.

Exclusion criteria: (1) Prior iodine-131 radioiodine therapy; (2) Preoperative imaging evidence of lateral cervical lymph node metastasis; (3) Distant metastasis detected before surgery; (4) History of prior thyroid surgery or head and neck radiotherapy; (5) Confirmed familial thyroid cancer or multiple endocrine neoplasias.

The standard diagnostic criteria for HT required the presence of four histopathological features on postoperative paraffin sections: diffuse lymphocytic infiltration involving ≥ 10% of the thyroid parenchyma, formation of lymphoid follicles, degeneration and atrophy of thyroid follicular epithelium, and eosinophilic change in the follicular epithelial cells. Cases with focal lymphocytic infiltration involving less than 10% of the tissue were excluded.

All surgical procedures for PTC in this study strictly adhered to the “Chinese Guidelines for the Diagnosis and Treatment of Thyroid Nodules and Differentiated Thyroid Carcinoma (2023 Edition)” (18), and surgical strategies were formulated according to standard risk-adapted principles. All participants received either thyroid lobectomy or total thyroidectomy combined with systematic central lymph node dissection at our center. All operations were carried out by two senior chief physicians of our department. Both surgeons have more than 15 years of experience in endocrine surgery, and each completes over 600 thyroid procedures every year. As a regional key specialty of thyroid surgery in South China, our department performs more than 1800 thyroid and parathyroid operations annually, ranking among the high-volume endocrine surgery centers nationwide.

### 2.2 Observation indices

#### 2.2.1 Data sources

Relevant clinical data were retrieved from the hospital electronic medical record system. Collected information included discharge summaries, admission records, perioperative laboratory and imaging results, operative notes, and postoperative pathological reports.

#### 2.2.2 Variable definitions

General clinical parameters: Variables included age, sex, body mass index (BMI), and length of hospital stay.

Laboratory indices: Detected biomarkers covered thyroid-stimulating hormone (TSH), thyroid peroxidase antibody (TPOAb), and thyroglobulin antibody (TgAb). All assays were interpreted referring to the reference ranges established by our institution. The normal TSH level was 0.56–5.91 μIU/mL; subnormal values indicated hyperthyroidism, while elevated levels suggested hypothyroidism, and any out-of-range result was deemed abnormal. The reference range was 0–9 IU/mL for TPOAb and 0–115 IU/mL for TgAb, with readings above the upper limits defined as seropositive. The normal range of thyroglobulin (Tg) was 0.00–30.00 ng/mL, and that of parathyroid hormone (PTH) was 1.6–6.9 pmol/L; values outside the respective ranges were regarded as abnormal. Hyperthyroidism was diagnosed when low TSH was accompanied by elevated free thyroxine (FT4) and free triiodothyronine (FT3). Hypothyroidism was defined as elevated TSH combined with decreased FT4 and FT3. Postoperative hypoparathyroidism was identified if serum PTH measured on postoperative day 1 fell below the lower limit of the laboratory reference range.

Pathological characteristics: Assessed parameters included tumor size, tumor location, multifocality, extrathyroidal extension, total retrieved lymph nodes, metastatic lymph nodes, and presence of lymphocytic thyroiditis. Tumor size was stratified by 1 cm as the cutoff value, which follows the standard classification of papillary thyroid microcarcinoma defined by the 2023 Chinese Thyroid Cancer Guidelines and global consensus. Stratification was performed as follows: tumor size (≤ 1 cm/> 1 cm), tumor location (left/right/bilateral/isthmus), multifocality (present/absent), and extrathyroidal extension (present/absent). The CLNM rate was calculated as (cases with positive central lymph nodes/total cases) × 100%. For patients with confirmed CLNM, the metastatic lymph node ratio was computed as (metastatic lymph nodes/total retrieved lymph nodes) × 100%. Multifocality was defined as the presence of two or more lesions. Extrathyroidal extension was defined as tumor penetration through the thyroid capsule into adjacent soft tissues.

Prognostic indicators: All patients were staged according to the 8th edition AJCC thyroid cancer staging system (stages I–IV). Postoperative recurrence risk was stratified according to the 2023 Guidelines for the Diagnosis and Treatment of Thyroid Nodules and Differentiated Thyroid Cancer (2nd Edition) (18), including low-risk, intermediate-risk, and high-risk groups.

### 2.3 Group assignment

**Figure 1:**
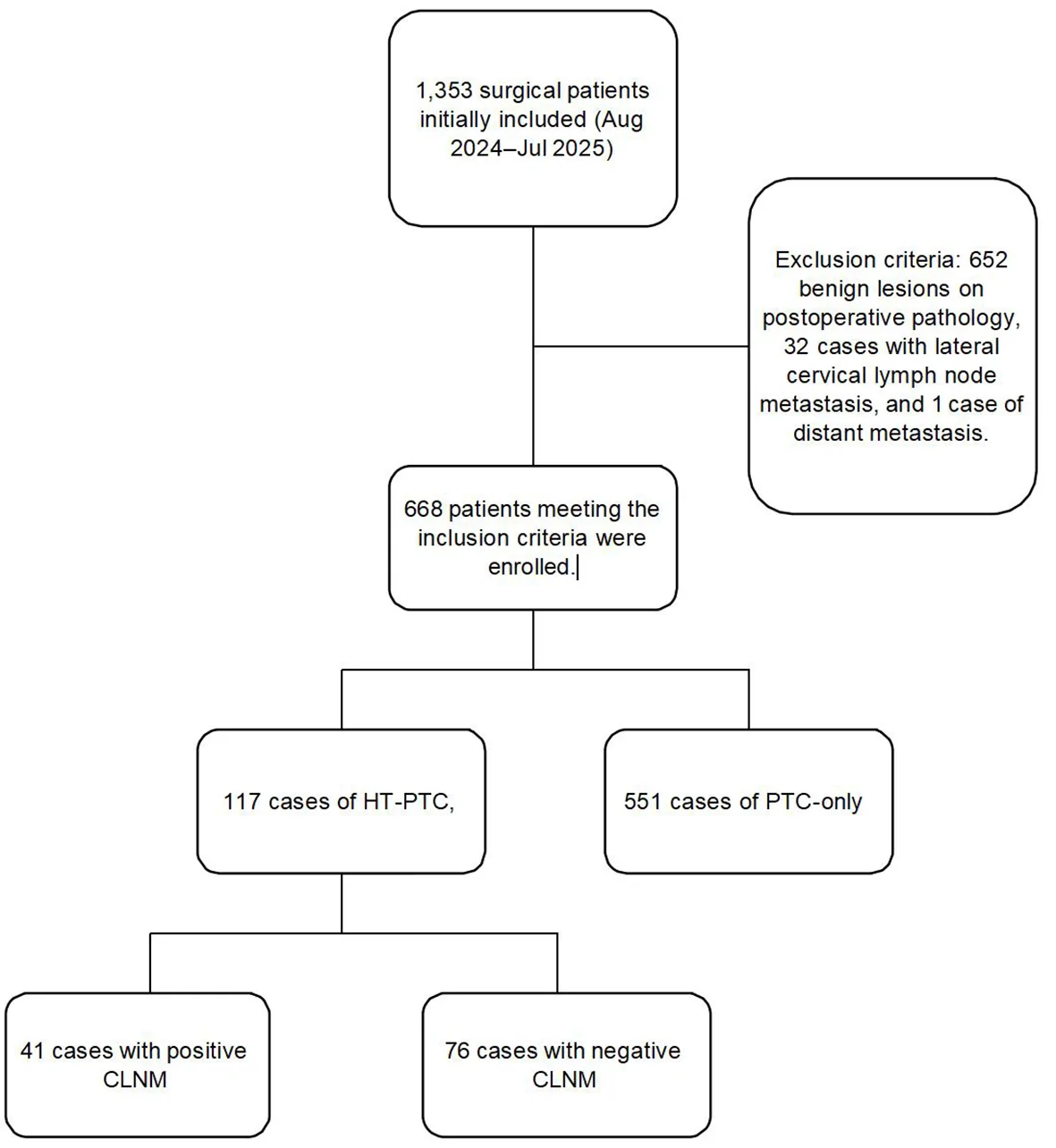
Case selection flowchart.

The 668 patients with PTC were categorized into two cohorts: the HT-PTC group (n = 117) and the PTC-only group (n = 551), based on the presence or absence of concomitant HT. The HT-PTC cohort was further classified into CLNM-positive (n = 41) and CLNM-negative (n = 76) subgroups. The detailed grouping strategy is illustrated in **Figure 1**.

### 2.4 Statistical analysis

Statistical analyses were performed using SPSS 26.0. The Shapiro–Wilk test was applied to assess the normality of continuous variables. A *P* value > 0.05 indicated a normal distribution. Normally distributed data were presented as mean ± standard deviation and compared using the independent samples t-test. Non-normally distributed data were described using median (interquartile range), with between-group differences evaluated by the Mann–Whitney U test. Categorical variables were summarized as frequencies and percentages, and comparisons were performed using the Pearson χ² test or Fisher’s exact test. Variables with *P* < 0.1 in univariate analysis, along with clinically recognized risk factors for CLNM, were entered into a multivariate binary logistic regression model using the enter method. Odds ratios (OR) and 95% confidence intervals (95% CI) were calculated. A *P* value < 0.05 was considered statistically significant.

## 3 Results

### 3.1 Comparison of baseline characteristics between the HT-PTC group and PTC-only group

#### 3.1.1 General clinical profiles

In the HT-PTC cohort, patients were aged 18 to 80 years, with a mean age of 44.68 ± 12.49 years. The PTC-only group had an age range of 16 to 75 years, with a mean age of 44.60 ± 11.08 years; no significant difference in age distribution was observed (*P* = 0.955). The HT-PTC group comprised 105 females (89.7%) and 12 males (10.3%), whereas the PTC-only group included 410 females (74.4%) and 141 males (25.6%). Females accounted for a significantly larger proportion in the HT-PTC group (*P* < 0.001). The mean BMI was 23.24 ± 3.25 kg/m² in the HT-PTC group and 24.06 ± 3.51 kg/m² in the PTC-only group, indicating a lower BMI in the HT-PTC group (*P* = 0.021).

#### 3.1.2 Laboratory indices

The median thyroid-stimulating hormone (TSH) level was 2.21 (1.47, 3.20) μIU/mL in the HT-PTC cohort, significantly higher than the 1.58 (1.07, 2.43) μIU/mL observed in the PTC-only group (*Z* = -4.334, *P* < 0.001). The prevalence of hypothyroidism was 5.1% in the HT-PTC group versus 1.1% in the PTC-only group (*P* = 0.009). No significant difference was observed in the prevalence of hyperthyroidism (0.9% vs. 2.9%, *P* = 0.340). Abnormal TSH levels were detected in 12 (10.3%) patients in the HT-PTC group and 53 (9.6%) patients in the PTC-only group (*P* = 0.833). The HT-PTC group demonstrated a significantly higher positive rate of thyroid peroxidase antibody (TPOAb) (61.5% vs. 18.3%, *P* < 0.001). Median TPOAb levels were 33.20 (1.70, 318.95) IU/mL and 0.70 (0.30, 3.70) IU/mL for the respective groups (*Z* = -9.548, *P* < 0.001). A total of 103 patients (88.0%) in the HT-PTC group had thyroglobulin (Tg) levels below 30 ng/mL, a proportion higher than the 78.2% (431 cases) observed in the PTC-only group (*P* = 0.016). The median Tg level was 4.86 (1.16, 14.71) ng/mL in the HT-PTC group, which was lower than the 12.87 (5.32, 26.63) ng/mL in the PTC-only group (*Z* = -5.500, *P* < 0.001).

Regarding PTH parameters, the mean preoperative PTH was 4.59 ± 1.54 pmol/L in the HT-PTC group and 4.44 ± 1.77 pmol/L in the PTC-only group, showing no significant difference (*P* = 0.404). Mean postoperative PTH was 2.59 ± 1.25 pmol/L and 2.61 ± 1.44 pmol/L, respectively (*P* = 0.903). The incidence of postoperative hypoparathyroidism was 27.4% (32/117) and 24.5% (135/551), with no significant intergroup difference (*P* = 0.518).

#### 3.1.3 Pathological characteristics

The median maximum tumor diameter was 8.00 (5.00, 13.50) mm in the HT-PTC cohort versus 7.00 (4.00, 10.00) mm in the PTC-only group (*Z* = -2.293, *P* = 0.022). However, both cohorts showed comparable rates of papillary thyroid microcarcinoma (PTMC) (≤ 1 cm) (*P* = 0.103). Bilateral lesions were identified in 19 (16.2%) patients of the HT-PTC group and 63 (11.4%) patients of the PTC-only group (*P* = 0.150). Multifocal tumors occurred in 34 (29.1%) HT-PTC patients, a proportion significantly higher than the 20.1% (111 cases) in the PTC-only cohort (*P* = 0.034). The rate of extrathyroidal extension was similar between cohorts (24.8% vs. 26.0%, *P* = 0.793) (**Table 1**).

**Table 1:** Comparison of baseline characteristics between the HT-PTC group and the PTC-only group.

Variable	HT-PTC	PTC-only	*χ2/t/Z*	*P* value
Age	44.68 ± 12.49	44.60 ± 11.08	0.057	0.955
Sex			12.85	< 0.001
Male	12 (10.3%)	141 (25.6%)		
Female	105 (89.7%)	410 (74.4%)		
Length of hospital stay	5.36 ± 1.68	5.43 ± 1.71	0.388	0.698
BMI	23.24 ± 3.25	24.06 ± 3.51	2.314	0.021
TSH, μIU/mL	2.21 (1.47, 3.20)	1.58 (1.07, 2.43)	-4.334	0.001
Hyperthyroidism			0.912	0.340
Yes	1 (0.9%)	16 (2.9%)		
No	116 (99.1%)	535 (97.1%)		
Hypothyroidism			6.783	0.009
Yes	6 (5.1%)	6 (1.1%)		
No	111 (94.9%)	545 (98.9%)		
TPOAb, IU/mL	33.20 (1.70, 318.95)	0.70 (0.30, 3.70)	-9.548	< 0.001
TPOAb positivity			93.885	< 0.001
Yes	72 (61.5%)	101 (18.3%)		
No	45 (38.5%)	450 (81.7%)		
Tg	4.86 (1.16, 14.71)	12.87 (5.32, 26.63)	-5.5	< 0.001
Tg < 30 ng/mL			5.795	< 0.016
Yes	103 (88.0%)	431 (78.2%)		
No	14 (12.0%)	120 (21.8%)		
Preoperative PTH	4.59 ± 1.54	4.44 ± 1.77	0.836	0.404
Postoperative PTH	2.59 ± 1.25	2.61 ± 1.44	0.122	0.903
Postoperative hypoparathyroidism			0.418	0.518
Yes	32 (27.4%)	135 (24.5%)		
No	85 (72.6%)	416 (75.5%)		
Tumor location			Fisher	0.300
Left	41 (35.0%)	227 (41.2%)		
Right	56 (47.9%)	248 (45.0%)		
Bilateral	19 (16.2%)	63 (11.4%)		
Isthmus	1 (0.9%)	13 (2.4%)		
Tumor Size	8.00 (5.00, 13.50)	7.00 (4.00, 10.00)	-2.293	0.022
Tumor size ≤ 1 cm			2.661	0.103
Yes	81 (69.2%)	421 (76.4%)		
No	36 (30.8%)	130 (23.6%)		
Extrathyroidal extension			0.069	0.793
Yes	29 (24.8%)	143 (26.0%)		
No	88 (75.2%)	408 (74.0%)		
Multifocality			4.513	0.034
Yes	34 (29.1%)	111 (20.1%)		
No	83 (70.9%)	440 (79.9%)		
No. of central LNs dissected	8.00 (5.00, 11.00)	5.00 (2.00, 7.00)	-7.285	<0.001
No. of metastatic lymph nodes	0.00 (0.00, 1.00)	0.00 (0.00, 1.00)	-0.033	0.973
Lymph node metastasis			0.052	0.819
Yes	41 (35.0%)	187 (33.9%)		
No	76 (65.0%)	364 (66.1%)		
Stage				0.200
Recurrence risk stratification				0.971

### 3.2 Comparison of central lymph node profiles between HT-PTC and PTC-only groups

The overall rate of CLNM was 35.0% (41/117) in the HT-PTC cohort versus 33.9% (187/551) in patients with PTC-only; no significant disparity was detected (χ² = 0.052, *P* = 0.819).

Regarding lymph node counts, the median number of harvested central lymph nodes was 8.00 (5.00, 11.00) in the HT-PTC group, significantly higher than the 5.00 (2.00, 7.00) in the PTC-only group (*Z* = -7.285, *P* < 0.001). Both groups presented a median metastatic lymph node count of 0.00 (0.00, 1.00), with no significant difference (*Z* = -0.033, *P* = 0.973).

The metastatic lymph node ratio (LNR) was analyzed in patients with LNR > 0. Among 41 CLNM-positive HT-PTC patients, 394 central lymph nodes were retrieved, with 119 harboring metastatic lesions, yielding a median LNR of 22.22% (14.84, 50.00). In the PTC-only group, 187 CLNM-positive individuals had 1338 nodes retrieved, with 619 metastatic foci, resulting in a median LNR of 50.00% (25.00, 70.00). The groups differed significantly in LNR (*Z* = -3.465, *P* < 0.001) (**Table 2**).

**Table 2:** Comparison of central lymph node characteristics between the two groups.

Variable	HT-PTC	PTC-only	*χ2/Z*	*P* value
Central lymph node metastasis rate	35.0% (41/117)	33.9% (187/551)	*χ²* = 0.052	0.819
Number of central lymph nodes dissected	8.00 (5.00, 11.00)	5.00 (2.00, 7.00)	*Z = -*7.285	< 0.001
Number of metastatic lymph nodes	0.00 (0.00, 1.00)	0.00 (0.00, 1.00)	*Z = -*0.033	0.973
Total dissected lymph nodes in CLNM-positive cases	394	1, 338	-	-
Total metastatic lymph nodes in CLNM-positive cases	119	619	-	-
Metastatic LN ratio	22.22 (14.84, 50.00)	50.00 (25.00, 70.00)	*Z* = -3.465	< 0.001

Subgroup analysis restricted to PTMC (≤ 1 cm) revealed comparable CLNM rates in the HT-PTC and PTC-only cohorts: 25.9% (21/81) versus 27.1% (114/421) (*P* = 0.830). Within this size-matched subgroup, patients with HT-PTC exhibited a significantly lower LNR (20.00% vs. 47.22%, *Z* = -3.387, *P* < 0.001).

Among 81 HT-PTC patients harboring lesions ≤ 1 cm without high-risk pathological features, the CLNM rate was 25.9% (21/81), significantly lower than the 55.5% recorded in patients with tumors > 1 cm (20/36, *P* = 0.002).

### 3.3 Comparison of prognostic markers between the two cohorts

Within the HT-PTC cohort, 112 patients (95.7%) were classified as clinical stage I and 5 (4.3%) as stage II. For PTC-only patients, 539 (97.8%) were in stage I, 7 (1.3%) in stage II, and 5 (0.9%) in stage III. The distribution of AJCC clinical stages showed no significant difference (*P* = 0.200).

Regarding postoperative recurrence risk stratification, 79 (67.5%) HT-PTC patients were classified as low-risk, 35 (29.9%) as intermediate-risk, and 3 (2.6%) as high-risk. Among PTC-only subjects, low-risk, intermediate-risk, and high-risk subgroups accounted for 68.1% (375 cases), 28.3% (156 cases), and 3.6% (20 cases), respectively. No significant discrepancy was observed in the distribution of recurrence risk grades across the two groups (*P* = 0.971).

### 3.4 Risk factor analysis for central lymph node metastasis among HT-PTC patients

#### 3.4.1 Univariate logistic regression

Univariate logistic regression analysis of HT-PTC patients identified tumor diameter > 1 cm as a significant correlate of CLNM (OR = 3.571; 95% CI: 1.562–8.142, *P* = 0.002). No significant associations were observed for age, sex, BMI, TSH, TPOAb, Tg, PTH, tumor location, extrathyroidal extension, or multifocality (all *P* > 0.05) (**Table 3**).

**Table 3:** Univariate logistic regression predictors of CLNM among HT-PTC patients.

Variable	CLNM(+)	CLNM(-)	*P* value	OR (Exp(B))	95% CI Lower - Upper
Age ≤ 55 years			0.712	0.844	0.342 - 2.083
Yes	32 (78.0%)	57 (75.0%)			
No	9 (22.0%)	19 (25.0%)			
Sex			0.613	0.730	0.216 - 2.465
Male	5 (12.2%)	7 (9.2%)			
Female	36 (87.8%)	69 (90.8%)			
BMI ≤ 24			0.391	1.400	0.649 - 3.019
Yes	22 (53.7%)	47 (61.8%)			
No	19 (46.3%)	29 (38.2%)			
Hyperthyroidism			0.999	–	–
Yes	0 (0.0%)	1 (1.3%)			
No	41 (100.0%)	75 (98.7%)			
Hypothyroidism			0.352	2.817	0.318 - 24.962
Yes	1 (2.4%)	5 (6.6%)			
No	40 (97.6%)	71 (93.4%)			
TSH abnormality			0.613	0.730	0.216 - 2.465
Yes	5 (12.2%)	7 (9.2%)			
No	36 (87.8%)	69 (90.8%)			
TPOAb positivity			0.927	1.037	0.476 - 2.262
Yes	25 (61.0%)	47 (61.8%)			
No	16 (39.0%)	29 (38.2%)			
Tg ≤ 30			0.955	1.034	0.322 - 3.318
Yes	36 (87.8%)	67 (88.2%)			
No	5 (12.2%)	9 (11.8%)			
Postoperative hypoparathyroidism			0.733	0.863	0.371 - 2.008
Yes	12 (29.3%)	20 (26.3%)			
No	29 (70.7%)	56 (73.7%)			
Tumor location			0.998	–	–
Left	18 (43.9%)	23 (30.3%)			
Right	13 (31.7%)	43 (56.6%)			
Bilateral	9 (22.0%)	10 (13.2%)			
Isthmus	1 (2.4%)	0 (0.0%)			
Tumor size ≤ 1 cm			0.002	3.571	1.567 - 8.142
Yes	21 (51.2%)	60 (78.9%)			
No	20 (48.8%)	16 (21.1%)			
Extrathyroidal extension			0.205	0.574	0.243 - 1.355
Yes	13 (31.7%)	16 (21.1%)			
No	28 (68.3%)	60 (78.9%)			
Multifocality			0.696	1.184	0.508 - 2.760
Yes	11 (26.8%)	23 (30.3%)			
No	30 (73.2%)	53 (69.7%)			

#### 3.4.2 Multivariate logistic regression analysis

Based on the Events Per Variable (EPV) principle, clinically recognized factors, including sex, multifocality, and age, were incorporated into the multivariate model. The Hosmer–Lemeshow test confirmed model goodness-of-fit. Multivariate analysis identified tumor diameter > 1 cm as an independent predictor of CLNM in HT-PTC patients (OR = 3.678; 95% CI: 1.587–8.520, *P* = 0.002). Sex, multifocality, and age did not demonstrate independent correlation (all *P* > 0.05) (**Table 4**).

**Table 4:** Results of multivariate logistic regression analysis for CLNM in HT-PTC patients.

Predictor variable	*B*	*SE*	Wald	df	*P* value	OR (Exp(B))	95% CI Lower - Upper
Sex	-0.253	0.66	0.147	1	0.702	0.777	0.213 - 2.833
Tumor size > 1 cm	1.302	0.429	9.229	1	0.002	3.678	1.587 - 8.520
Multifocality	0.028	0.458	0.004	1	0.951	1.029	0.419 - 2.524
Age ≤ 55 years	-0.384	0.498	0.594	1	0.441	0.681	0.257 - 1.808
Constant	0.071	0.511	0.019	1	0.890	1.074	

#### 3.4.3 Development and performance validation of the predictive model

**Figure 2:**
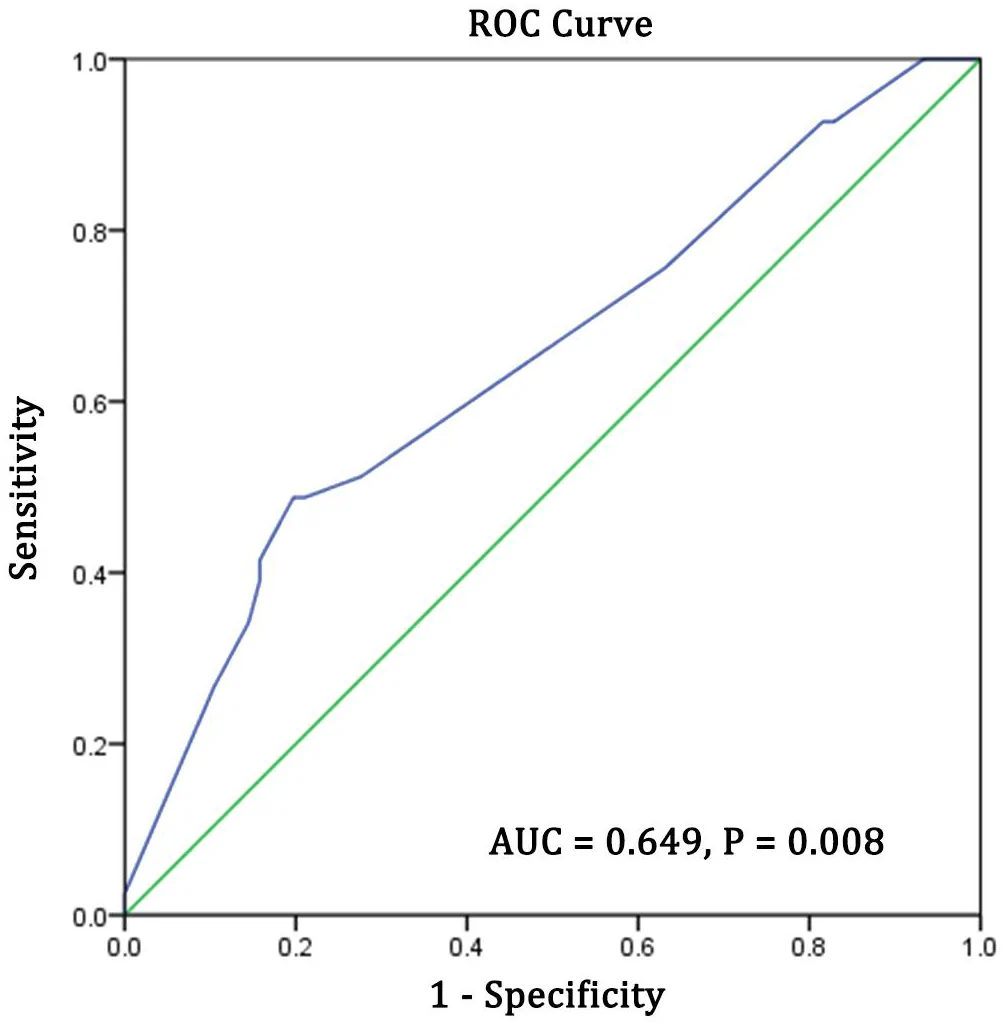
ROC curve for predicting the risk of CLNM among patients with HT-PTC.

A predictive model for CLNM was developed based on the multivariate logistic regression findings. The Hosmer–Lemeshow test (χ² = 1.185, df = 4, *P* = 0.881) demonstrated favorable calibration. The predictive model demonstrated a moderate discriminatory ability, yielding an AUC of 0.649 (95% CI: 0.543–0.755, *P* = 0.008), which was significantly better than random chance. The optimal probability cut-off, determined by the maximum Youden index (0.291), was 0.521, with a sensitivity of 48.8% and specificity of 80.3%. The positive predictive value and negative predictive value were 58.3% and 75.3%, respectively. Validation was restricted to internal testing relying solely on the 117 HT-PTC subjects within this single cohort, and no independent external dataset was available for external validation. The model could serve as a preliminary tool for preoperative risk stratification of CLNM in patients with HT-PTC (**Figure 2**).

## 4 Discussion

### 4.1 Baseline clinical and laboratory profiles in patients with concurrent HT and PTC

Gender Profiles: Our cohort data indicated that females accounted for 89.7% of the HT-PTC group, significantly higher than the 74.4% recorded in the PTC-only group (*P* < 0.001). This finding aligns with multiple prior cohort investigations (15, 19, 20). Estrogen acts as a potent mitogen and immune regulator via ERα/ERβ on thyroid follicular cells: it boosts thyroid epithelial and tumor cell proliferation, upregulates CCND1 and anti-apoptotic Bcl-2, and elevates thyroid autoantibodies (21–23). This dual regulatory effect largely explains the female predominance observed in HT-PTC patients.

Metabolic Profiles: Patients with HT-PTC exhibited a lower mean BMI compared with the PTC-only group (23.24 vs. 24.06 kg/m², *P* = 0.021). Although some literature suggests HT populations may carry higher risks of metabolic derangements (24), the lower BMI observed here warrants further exploration. Potential contributors include the limited sample size and the possibility that autoimmune-driven chronic inflammation may increase resting energy expenditure (25). Prospective investigations are necessary to validate these observations.

Serological Profiles: HT-PTC patients displayed a median TSH of 2.21 (1.47, 3.20) μIU/mL, higher than the 1.58 (1.07, 2.43) μIU/mL in the PTC-only group (*Z* = -4.334, *P* < 0.001). Increased rates of hypothyroidism (5.1% vs. 1.1%, *P* = 0.009) and TPOAb seropositivity (61.5% vs. 18.3%, *P* < 0.001) were also observed, consistent with the immune phenotype of HT (26, 27). Cumulative evidence suggests that HT-triggered lymphocytic infiltration and follicular disruption impair hormone biosynthesis, triggering sustained TSH elevation via negative feedback. Chronic TSH overstimulation drives follicular proliferation and genomic instability, amplifying malignant transformation susceptibility—the central endocrine cascade linking HT to PTC tumorigenesis (28, 29). Notably, our cohort further documented a markedly lower median Tg level of 4.86 (1.16, 14.71) ng/mL among HT-PTC patients, versus 12.87 (5.32, 26.63) ng/mL in PTC-only cases (*Z* = -5.500, *P <*0.001). This observation reproduces the findings published in Zheng’s prospective cohort research (27), reflecting glandular destruction and atrophy secondary to chronic inflammation (30). Regarding surgical outcomes, no intergroup differences were observed in pre- or postoperative PTH levels or the incidence of permanent hypoparathyroidism. This suggests that while HT imposes technical challenges during dissection, it does not independently increase the risk of permanent hypoparathyroidism (31–33). Provided that standardized surgical protocols are maintained and parathyroid glands are meticulously preserved, no additional prophylactic measures appear necessary solely for patients with concurrent HT.

### 4.2 Effects of HT on pathological characteristics and CLNM in PTC patients

The most prominent observation from our cohort illustrates the dual regulatory effects exerted by HT on the invasive phenotypes of PTC, characterized by a combination of elevated multifocal tumor prevalence and diminished lymph node metastatic burden. Our statistical comparison revealed a higher rate of multifocal lesions among patients with HT-PTC relative to those with PTC-only (29.1% vs. 20.1%, *P =* 0.034), implying that autoimmune thyroiditis facilitates multicentric tumorigenesis of papillary thyroid carcinoma. This finding aligns with outcomes from a large-scale meta-analysis covering over 40, 000 to 50, 000 PTC cases, which reported an elevated odds ratio of 1.17 for multifocal neoplasms in patients coexisting with HT (14). Mechanistically, the chronic inflammatory microenvironment from HT drives multifocal tumorigenesis. Specifically, diffuse lymphocyte infiltration, along with cytokines (TNF-α, IL-6) and reactive oxygen species, induces repeated thyroid epithelial injury and repair. This regenerative process activates NF-κB and cooperates with BRAF/RET mutations to ultimately promote PTC progression (12, 34). In parallel, patients suffering from HT frequently present with elevated circulating TSH concentrations. Beyond its canonical endocrine function, TSH acts as a mitogen for thyroid follicular epithelia and further fuels the proliferation of transformed cell clones (35). Nevertheless, no intergroup disparity in extrathyroidal extension rates was detected between the two cohorts (24.8% vs. 26.0%, *P =* 0.793). Despite a numerically higher proportion of bilateral lesions recorded in the HT-PTC subgroup, this difference failed to reach statistical significance (16.2% vs. 11.4%, *P =* 0.15). Collectively, these data indicate that HT predominantly modulates the risk of multifocal tumor formation rather than altering local invasive capacity of PTC lesions.

The median tumor size in the HT-PTC group was 8.00 (5.00, 13.50) mm, compared to 7.00 (4.00, 10.00) mm in the PTC-only group, with a statistically significant intergroup difference (*Z* = -2.293, *P =* 0.022). Nevertheless, the percentage of PTMC showed no meaningful intergroup discrepancy, which conflicts with findings reported in several prior publications (13, 26). Such inconsistent observations can be attributed to the multifaceted regulatory effects of HT on neoplastic biological phenotypes. For one thing, autoimmune inflammation triggered by HT remodels the local intratumoral inflammatory niche, driving enlarged lesion volume and higher multifocal incidence. For another, existing research demonstrates that despite larger primary tumor size among HT carriers, invasive markers including lymphatic metastasis and extrathyroidal extension, remain less prominent. This phenomenon implies HT could partially restrain aggressive tumor progression (14).

The correlation between HT and CLNM in PTC remains a widely debated research topic across endocrine surgery communities. Our cohort detected comparable overall CLNM prevalence between the two cohorts (35.0% vs. 33.9%, *P =* 0.819), consistent with a series of prior single-center retrospective studies. Such observations indicate HT does not serve as an independent risk determinant for CLNM among PTC patients (20, 36, 37). Our core novel finding lies in the divergent nodal metrics between subgroups despite equivalent CLNM positivity rates. Patients in HT-PTC yielded a median total harvested lymph node count of 8.00 (5.00, 11.00), significantly higher than the 5.00 (2.00, 7.00) retrieved from PTC-only cases (*Z* = -7.285, *P <*0.001). By contrast, both cohorts shared an identical median metastatic node count of 0.00 (0.00, 1.00), without detectable intergroup divergence (*Z* = -0.033, *P =* 0.973). This contrast corresponded to a lower median LNR observed in the HT-PTC subgroup (22.22% vs. 50.00%, *Z* = -3.465, *P <* 0.001). Stratified analysis confined to PTMC (tumor size ≤ 1 cm) reproduced this trend: after matching the tumor diameter baseline, the HT-PTC subgroup still displayed suppressed LNR values (20.00% vs. 47.22%, *Z* = -3.387, *P <* 0.001). Mechanistically, HT-driven persistent chronic inflammation triggers reactive lymphadenopathy in the central neck compartment, expanding the pool of lymph nodes identifiable and resectable during thyroidectomy. Meanwhile, the HT-primed autoimmune microenvironment may restrain malignant cell invasion and colonization in lymph nodes (38), contributing to the reduced lymph node ratio (LNR). Since a reduced nodal metastatic burden is generally considered a surrogate for favorable outcomes (39), this finding supports individualized surgical decision-making for HT-PTC patients.

### 4.3 Analysis of risk factors for CLNM in patients with HT-PTC

Univariate followed by multivariate logistic regression modeling was performed to screen risk predictors in our cohort. The analyses identified maximum tumor diameter exceeding 1 cm as the only independent predictor of CLNM among HT-PTC patients (OR = 3.678; 95% CI: 1.587–8.520, *P* = 0.002), matching robust outcomes from abundant published clinical cohorts (6, 36, 40). Lesion size acts as a core biomarker reflecting the invasive potential of PTC. Larger tumor volume markedly elevates the likelihood of capsular penetration, lymphatic vascular invasion, and subsequent nodal dissemination. Even in patients with PTC complicated by HT, tumor size remains the most critical factor determining the risk of CLNM (41). This observation carries straightforward clinical implications. For HT-PTC individuals harboring lesions > 1 cm in diameter, thorough preoperative radiological assessment targeting central neck lymph nodes is mandatory. Intraoperatively, standardized systematic central compartment dissection ought to be conducted to minimize risks of postoperative residual tumor and long-term disease recurrence.

None of the covariates, including age, sex, BMI, serum TSH concentration, multifocality, and extrathyroidal extension, emerged as independent correlates of CLNM in our regression models. Two plausible explanations account for this negative finding. First, the overall sample size of the HT-PTC cohort was relatively constrained, with merely 41 CLNM-positive subjects enrolled, which compromised statistical power for subgroup stratification. Second, the HT-derived inflammatory niche may blunt the oncogenic influence exerted by conventional risk predictors on lymphatic metastatic spread. Large-scale multicenter investigations are warranted to verify the above deduction.

ROC curve analyses validated acceptable discriminative performance of this predictive model (AUC = 0.649). While the AUC did not reach an optimal threshold, the value retained statistical significance within this retrospective single-center dataset. This predictive model can assist clinicians in preoperative risk stratification of CLNM for HT-PTC patients to guide individualized surgical decision-making. External validation using separate patient cohorts is required to verify the stability of this predictive model.

### 4.4 Impact of HT on prognosis stratification in PTC patients

No intergroup disparities were detected regarding AJCC tumor staging and recurrence risk classification (*P =* 0.200, *P =* 0.971). Clinical stage I disease accounted for nearly all cases in both cohorts (95.7% for HT-complicated PTC vs. 97.8% for PTC-only), and the proportion of low-risk individuals was comparable across subgroups (67.5% vs 68.1%). These observations corroborate the notion that concomitant HT fails to modify baseline prognostic stratification of PTC. Nevertheless, several limitations should be acknowledged. As a retrospective single-center analysis, our assessment only relied on short-term postoperative pathological data without extended follow-up records, making it impossible to quantify how HT shapes 5-year and 10-year recurrence-free survival (RFS) as well as overall survival (OS). Cumulative large-scale international and domestic cohort investigations plus meta-analyses have demonstrated that PTC patients complicated with HT generally present smaller primary lesions, lighter lymphatic metastatic burden, rarer distant dissemination, markedly reduced long-term recurrence hazards, and superior disease-specific survival (DSS). Such findings imply a tumor-protective property conferred by HT (14, 26, 29). A multicenter cohort conducted by Marotta et al. validated HT as an independent favorable prognostic biomarker, which correlates with higher complete clinical remission rates and prolonged recurrence-free survival (42). Taken together, current evidence reveals a beneficial tendency of HT toward long-term oncologic outcomes of PTC. Further large-scale prospective trials with prolonged surveillance are required to elaborate its exact molecular mechanisms and corresponding clinical implications.

One core observation derived from our cohort demonstrates that patients with concurrent HT and PTC residing in coastal South China present a distinct clinicopathological phenotype defined by elevated multifocal tumor burden accompanied by diminished LNR. Despite classification as an iodine-sufficient zone overall, coastal residents across China exhibit lower urinary iodine concentrations compared with inland populations owing to distinct dietary patterns and nationwide universal salt iodization policies (43–45). Such a unique profile featuring abundant dietary iodine intake yet relatively low systemic iodine turnover may remodel the intrathyroidal oncogenic microenvironment via two divergent pathways. For one, regional relative iodine scarcity triggers physiological TSH elevation, consistent with our measurement of higher TSH concentrations among HT-complicated PTC patients versus PTC-only counterparts. Sustained TSH stimulation drives progressive thyroid follicular hyperplasia and ultimately raises the prevalence of multifocal lesions (29.1% vs 20.1%). For another, this region-specific iodine nutritional milieu may modulate the tumoricidal activity of HT-associated infiltrating lymphocytes to restrain lymphatic dissemination of malignant cells, which accounts for the reduced nodal metastatic burden. Collectively, these findings underscore the critical value of region-specific clinical investigations tailored to distinct iodine exposure backgrounds. Iodine status directly modulates the intensity of HT-mediated autoimmune inflammation and intrathyroidal anti-tumor immune activity; populations with iodine deficiency or excessive iodine intake may present disparate multifocality and metastatic lymph node ratio profiles. For this reason, the conclusions derived from our iodine-sufficient coastal South China cohort cannot be directly generalized to inland regions with different iodine nutritional conditions.

## 5 Conclusions

This study found no significant correlation between HT and increased CLNM prevalence in cN0 PTC patients, but noted a lower LNR in HT-PTC cases. For HT-PTC patients with tumors ≤ 1 cm and no other high-risk factors, the necessity of prophylactic central lymph node dissection warrants individualized assessment combining clinical data and intraoperative observations to avoid overtreatment. Conversely, for tumors > 1 cm, rigorous imaging and standardized systematic central compartment dissection are recommended to mitigate recurrence risk. This research provides evidence-based guidance for individualized PTC management in this region.

## 6 Study limitations

This study has certain limitations:

First, this retrospective single-cohort investigation was conducted at a single institution. The HT-PTC subgroup contained merely 117 cases, with only 41 CLNM-positive events, which may reduce the statistical power of multivariate logistic regression analysis and restrict the reliability of subgroup risk factor screening results. Additionally, only patients with standardized central lymph node dissection were included, which may bias CLNM incidence estimates; our results apply solely to cN0 PTC patients undergoing complete central compartment dissection. Individual urinary iodine measurements were not available, rendering the dataset susceptible to selection bias. Furthermore, the confounding effect of varied surgical approaches on retrieved nodal counts remained unadjusted, which may bring residual confounding bias. Given the region-specific iodine nutritional profile, the extrapolation of our findings to territories with distinct iodine intake backgrounds is constrained. Large-scale multicenter cohorts are required in subsequent research to corroborate our core observations.

Second, key molecular drivers governing PTC malignant phenotypes, including BRAF V600E and TERT promoter mutations, were not incorporated into our analytical framework. Accordingly, the interactive regulatory crosstalk among molecular signatures, HT, and central lymph node metastasis could not be deciphered. Further mechanistic exploration, combined with molecular laboratory assays, is warranted to address this gap. Notably, the CLNM prediction model was only validated internally within the HT-PTC subgroup without external independent cohorts, so its generalizability to other medical centers remains unconfirmed; multicenter external validation is urgently needed in future studies.

Third, our analysis exclusively focused on the metastatic status of central compartment nodes, without incorporating clinical data concerning lateral neck lymphatic dissemination. HT-induced reactive lymphadenopathy increases the number of harvested central lymph nodes and may artificially decrease LNR; we mitigated this bias via LNR normalization and stratified microcarcinoma subgroup analysis. Additionally, long-term surveillance records were absent, making it impossible to quantify how HT modulates long-term postoperative recurrence risks, disease-free survival, and overall survival.

## Data Availability

The original contributions presented in the study are included in the article/supplementary material. Further inquiries can be directed to the corresponding author.
